# The multikinase inhibitor Sorafenib displays significant antiproliferative effects and induces apoptosis via caspase 3, 7 and PARP in B- and T-lymphoblastic cells

**DOI:** 10.1186/1471-2407-10-560

**Published:** 2010-10-15

**Authors:** Catrin Schult, Meike Dahlhaus, Sabine Ruck, Mandy Sawitzky, Francesca Amoroso, Sandra Lange, Daniela Etro, Aenne Glass, Georg Fuellen, Sonja Boldt, Olaf Wolkenhauer, Luca Maria Neri, Mathias Freund, Christian Junghanss

**Affiliations:** 1 University of Rostock, Division of Medicine, Department of Hematology/Oncology, Rostock, Germany; 2 University of Ferrara, Section of Human Anatomy, Department of Morphology and Embryology, Ferrara, Italy; 3 University of Rostock, Institute for Biostatistics and Informatics in Medicine and Ageing Research, Rostock, Germany; 4 University of Rostock, Department of Computer Science, Systems Biology and Bioinformatics, Rostock, Germany

## Abstract

**Background:**

Targeted therapy approaches have been successfully introduced into the treatment of several cancers. The multikinase inhibitor Sorafenib has antitumor activity in solid tumors and its effects on acute lymphoblastic leukemia (ALL) cells are still unclear.

**Methods:**

ALL cell lines (SEM, RS4;11 and Jurkat) were treated with Sorafenib alone or in combination with cytarabine, doxorubicin or RAD001. Cell count, apoptosis and necrosis rates, cell cycle distribution, protein phosphorylation and metabolic activity were determined.

**Results:**

Sorafenib inhibited the proliferation of ALL cells by cell cycle arrest accompanied by down-regulation of CyclinD3 and CDK4. Furthermore, Sorafenib initiated apoptosis by cleavage of caspases 3, 7 and PARP. Apoptosis and necrosis rates increased significantly with most pronounced effects after 96 h. Antiproliferative effects of Sorafenib were associated with a decreased phosphorylation of Akt (Ser473 and Thr308), FoxO3A (Thr32) and 4EBP-1 (Ser65 and Thr70) as early as 0.5 h after treatment. Synergistic effects were seen when Sorafenib was combined with other cytotoxic drugs or a mTOR inhibitor emphasizing the Sorafenib effect.

**Conclusion:**

Sorafenib displays significant antileukemic activity *in vitro *by inducing cell cycle arrest and apoptosis. Furthermore, it influences PI3K/Akt/mTOR signaling in ALL cells.

## Background

Acute lymphoblastic leukemias (ALL) can occur during childhood and more rarely during adulthood. Especially adult patients still have a grave prognosis following conventional chemotherapies despite progress in the treatment during recent years. Therefore, risk adapted therapy approaches have been developed including allogenic stem cell transplantation as well as targeted therapies. In particular, CD20 antibody treatment has been successfully introduced in B-ALL [1]. In addition, signal transduction inhibitors such as the tyrosine kinase inhibitor Imatinib have been used in BCR-ABL positive ALL patients leading to improved response rates [2,3]. Investigation of further targeted therapy approaches e.g. inhibition of signaling pathways is aiming at inhibiting other dysregulated tyrosine kinases or transcription factors.

Sorafenib is a multikinase inhibitor targeting Raf serine/threonine kinases as well as different receptor tyrosine kinases including c-Kit, FLT-3, vascular endothelial growth factor receptor (VEGFR) and platelet-derived growth factor receptor (PDGFR) [4,5]. Sorafenib has previously been shown to induce apoptosis and necrosis in various types of cancer such as renal cell carcinoma, breast cancer, lung cancer, colon cancer, chronic myelogenous leukemia (CML), chronic lymphocytic leukemia (CLL) and acute myeloid leukemia (AML) [6-8]. Cell lines from different solid tumors have been tested previously for their response to Sorafenib. It was shown that Sorafenib inhibits cell growth of renal cell carcinoma cells, pancreatic tumor cells, colon cancer, breast tumor cells and melanoma tumor cells [9,6]. Sorafenib has recently been approved for the clinical treatment of hepatocellular carcinoma and renal cell carcinoma [10]. Furthermore it is under clinical investigation in FLT3 positive acute myeloid leukemia (AML) patients [8,11]. In the present study we investigated the effect of the multikinase inhibitor Sorafenib on B- and T-ALL cells. Our results demonstrate that Sorafenib inhibits proliferation and induces apoptosis as well as necrosis in ALL cells. In addition, we could demonstrate the inhibitory effect of Sorafenib on the PI3K/Akt pathway.

## Methods

### Cell lines

ALL cell lines with different cytogenetics and phenotypes were used. The human ALL cell lines SEM, RS4;11 (both B-ALL) and Jurkat (T-ALL) were purchased from DSMZ (Braunschweig, Germany) and cultured according to manufacturer's protocol. The media were supplemented with 10% heat-inactivated fetal bovine serum (PAA, Pasching, Austria) and 1% penicillin and streptomycin (Biochrom AG, Berlin, Germany). All cells were grown in a 37°C and 5% CO_2 _humidified atmosphere-incubator.

### Inhibitors and cytostatics

Sorafenib was a kind gift from Bayer Healthcare (Leverkusen, Germany). The mTOR inhibitor RAD001 was kindly provided from Novartis (Basel, Switzerland). Inhibitors were dissolved in dimethyl sulfoxide (DMSO) and stored as stock solution at -20°C. Cytarabine and doxorubicin were purchased from cell pharm GmbH (Bad Vilbel, Germany) and dissolved in 5% NaCl (B. Braun Melsungen AG, Melsungen, Germany).

For experimental use drugs were prepared freshly from stock solution. Control cells were cultured in their medium containing the same concentration of DMSO as the experimental treated cells. Drug concentrations were chosen in accordance with serum concentration that can be achieved in clinical settings.

### Inhibition experiments and drug combination studies

Cells (5 × 10^5^/well) were seeded in 24 well plates (Nunc, Langenselbold, Germany) and treated with inhibitors for up to 96 h. Sorafenib was investigated as single drug and in combination with conventional cytostatics cytarabine and doxorubicin. In addition, the mTOR inhibitor RAD001 was combined with Sorafenib. Cells were incubated with sub -IC50 concentration of cytostatics cytarabine (250 nM), doxorubicin (25 nM) or RAD001 (1 nM and 10 nM) and with Sorafenib (0.73 μM and 7.3 μM) alone and in combination. Sub -IC50 concentrations of cytostatics were used to detect synergistics effects easier. IC-50 values of each drug had been determined in previous experiments. Inhibitors were added once at the time of cell seeding. Samples of cells were harvested after 0.5, 2.5, 4.0, 24, 48, 72 and 96 h and used for analyses.

### Analyses of apoptosis and necrosis

Apoptosis and necrosis were determined using Annexin V FITC (BD Biosciences, Heidelberg, Germany) and propidium iodide (PI) (Sigma Aldrich, St. Louis, USA) labeling technique and flow cytometry analyses. Briefly, 5 × 10^5 ^cells were harvested and washed twice (180 g, 10 min, 4°C) with PBS at indicated points in time. Each cell pellet was resuspended in 100 μl of binding buffer (1×) and 5 μl Annexin V FITC were added. After an incubation time of 10 min at room temperature, additional 400 μl of binding buffer were added for a final volume of 500 μl. Cells were stained with PI (0.6 μg/ml) immediately before measurement. Unstained and single stained controls were included in each experiment. Flow cytometry analyses were performed using FACSCalibur (Becton and Dickinson, Heidelberg, Germany) and data thus obtained were analysed with CellQuest software (Becton and Dickinson, Heidelberg, Germany).

### Proliferation studies

Cell counts were determined using the trypan blue staining. Metabolic activity was determined using tetrazolium compound (4-[3-(4-Iodophenyl)-2-(4-nitrophenyl)-2H-5-tetrazolio]-1,3-benzene disulfonate) [WST-1, Roche, Mannheim, Germany] according to the manufacturers protocol. In brief, cells (5 × 10^4^/well) were seeded in 96 well plates in triplicates and incubated with 15 μl WST-1 for 4 h. The assay is based on the reduction of tetrazolium salt WST-1 to soluble formazan by mitochondrial dehydrogenases of the cells. The amount of formazan dye directly correlates to the number of metabolically active cells and was detected by the absorbance at 450 nm and a reference wavelength at 620 nm by an ELISA Reader (Anthos, Krefeld, Germany). The absorbance of culture medium plus WST-1 in the absence of cells was used as background control.

### Cell cycle analysis

After treatment SEM and Jurkat cells were harvested and washed twice in PBS. Cells were fixed with 70% ethanol and incubated with 1 mg/ml Ribonuclease A (Sigma-Aldrich, St. Louis, USA) for 30 min at 37°C. Subsequently, cells were washed twice in PBS and stained with PI. DNA content was analyzed by flow cytometry on a FACSCalibur Cytometer (Becton and Dickinson, Heidelberg, Germany). Data analysis was performed using CellQuest software (Becton and Dickinson, Heidelberg, Germany).

### Western blot

For protein extraction 1 × 10^6 ^cells were washed twice in PBS and lysed with RIPA buffer (50 mM Tris HCl pH 7.4; 150 mM NaCl; 0.1% SDS and 1% NP40) including protease and phosphatase inhibitors (Roche Applied Science, Mannheim, Germany). Samples were incubated for 20 min at 4°C and frozen at -20°C. Cell extracts were thawed and centrifuged at 12000 g for 10 min at 4°C. Total protein concentration of supernatants was determined using Bio-Rad Protein Assay (Bio-Rad, München, Germany).

Equal amounts of protein samples were separated by SDS-polyacrylamid gel (8% or 15%) electrophoresis and transfered onto a PVDF membrane (Amersham Biosciences, Buckinghamshire, UK). Membranes were blocked in 5% milk or 5% BSA and incubated at 4°C overnight with the following polyclonal antibodies: rabbit anti-cleaved caspase 3, rabbit anti-caspase 3, rabbit anti-cleaved PARP, rabbit anti-cleaved caspase 7, rabbit anti-caspase 7, rabbit anti-pErk1/2 (Thr202/Tyr204), rabbit anti-Erk, rabbit anti-pAktThr308, rabbit anti-pAktSer473, rabbit anti-Akt, rabbit anti-p15^INKB^, rabbit anti-p27^KIP1^, mouse anti-CDK4, mouse anti-CyclinD3 (all Cell Signaling Frankfurt/Main, Germany), rabbit anti-pFoxO3AThr32 and rabbit anti-FoxO3A (both Upstate, Temecula, USA). Blots were incubated with mouse anti-α-tubulin antibody or mouse anti-GAPDH (both Invitrogen, Carlsbad, USA) as loading control. Specific horseradish peroxidase-conjugated secondary antibodies (anti-mouse or anti-rabbit) were used. Blots were re-probed using Restore Plus Western Blot stripping buffer (Pierce, Rockford, USA). Signals were detected with ECL Plus reagent (Amersham Biosciences; Buckinghamshire, UK) and a CCD camera (Kodak Digital Sience Image Station 440CF, Rochester, USA).

### Statistical analysis

Experiments were conducted in triplicates and results within each experiment were described using mean ± standard deviation (SD). Significant effects (*) between treatment groups, or between treatment groups and control was accomplished by using the two-sample Student's *t*-test. For more than two independent samples, the total significance level α = 0.05 was Bonferroni adjusted for each pairwise test (α_adj_). All p-values resulted from two-sided tests. The nature of interaction between Sorafenib and other drugs was characterized using Bliss additivism model [12-14].

## Results

### Sorafenib inhibits proliferation and induces apoptosis in ALL cells

**Figure 1:**
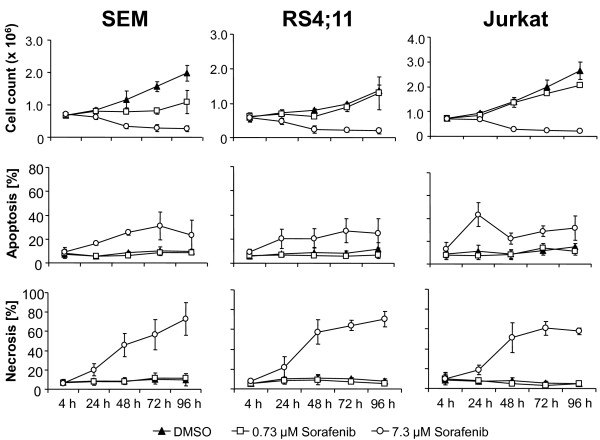
**Treatment with Sorafenib inhibits cell proliferation and induces apoptosis**. SEM, RS4;11 and Jurkat cells were exposed to the indicated concentrations of Sorafenib. The proliferation of SEM, RS4;11 and Jurkat are significantly inhibited, with most pronounced effects at 96 h. Apoptotic and necrotic cells were analyzed using Annexin V/PI staining followed by flow cytometry. Apoptosis and necrosis rates increased in B- and T-ALL cell lines following Sorafenib treatment. Results are displayed as mean +/- SD of three independent experiments.

The influence of the multikinase inhibitor Sorafenib on proliferation in ALL cell lines SEM, RS4;11 and Jurkat was analyzed. Cells were incubated with two different concentrations of Sorafenib (0.73 μM and 7.3 μM). Results are summarized in Figure 1. Cell proliferation of all investigated ALL cell lines was significantly inhibited at a Sorafenib concentration of 7.3 μM. Proliferation inhibition was seen as early as 24 h after first exposure. The most pronounced effects were achieved at 96 h. Treatment with 0.73 μM Sorafenib also inhibited the proliferation in SEM cells, but not in RS4;11 and Jurkat cells.

**Figure 2:**
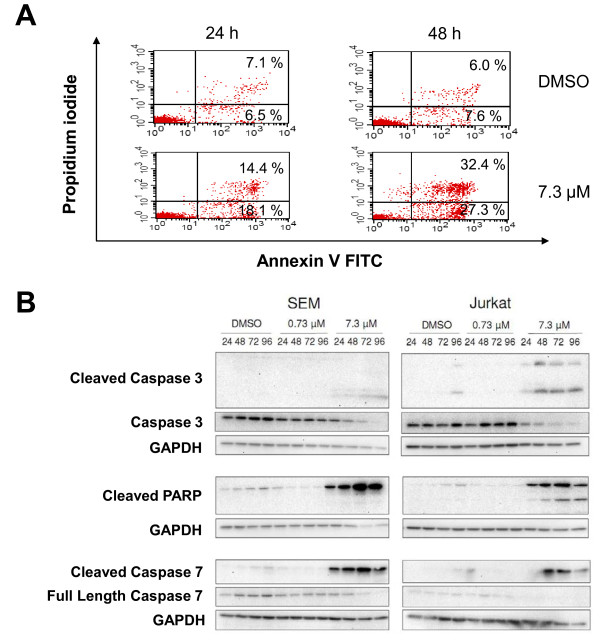
**Sorafenib induces apoptosis via cleavage of caspases**. **(A) **Apoptotic (Annexin V FITC^+ ^and Propidium iodide^-^) and necrotic cells (Annexin V FITC^+ ^and Propidium iodide^+^) were detected by flow cytometry analysis at 24 h and 48 h following Sorafenib treatment. Results are displayed for SEM cells treated with DMSO or 7.3 μM Sorafenib. **(B) **Cells were treated as described in Figure 1. Total cell lysates (25 μg) were analyzed by Western blot to detect the cleavage of Caspase 3, 7 and PARP. GAPDH was used as control for equal loading.

Sorafenib induced apoptosis and necrosis in ALL cells. Highest mean apoptosis and necrosis rates with 7.3 μM Sorafenib were 30.8%, 26.8%, 43.4% and 72.9%, 70.4%, 60.5% for SEM, RS4;11 and Jurkat, respectively. Analyses for apoptosis and necrosis using Annexin FITC and Propidiumiodid stainig are presented in Figure 2A. Dot plots are shown for SEM cells after Sorafenib exposure at 24 h and 48 h.

We then investigated the effects of Sorafenib on apoptosis induction in SEM and Jurkat cells in more detail. Treatment with Sorafenib induced apoptosis by cleavage of caspases 3, 7 and PARP that was observed already 24 h after treatment with 7.3 μM Sorafenib. Results are displayed in Figure 2.

### Sorafenib induces cell cycle arrest

**Figure 3:**
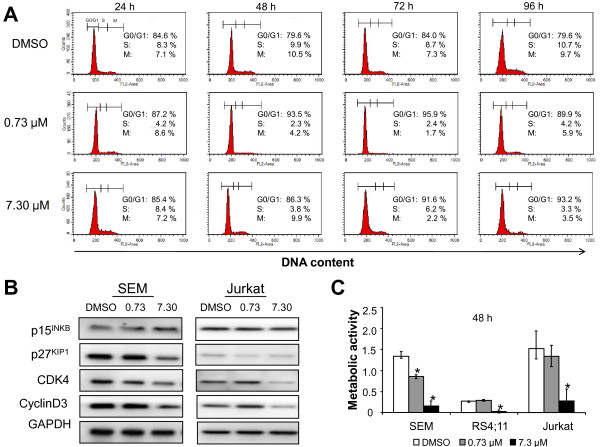
**Sorafenib inhibits cell cycle progression and metabolic activity**. **(A) **Effects of Sorafenib on cell cycle distribution was determined by DNA staining with Propidium iodide and flow cytometry in SEM cell line. Sorafenib induces a reduction of cells in M and S phases. **(B) **Cell cycle arrest was confirmed by western blot 24 h after Sorafenib treatment in SEM and Jurkat cells. Membranes were incubated with the indicated antibodies against cell cycle regulator proteins. GAPDH was used as control for equal loading. Reduction of CDK4 and Cyclin D3 were detected at 7.3 μM Sorafenib in both cell lines. **(C) **SEM, RS4;11 and Jurkat cells were treated with the indicated concentration of Sorafenib for 48 h and metabolic activity were analyzed using WST-1 Assay. Metabolic activity measured by formazan dye is shown as a difference in absorbance measured at 450 nm and 620 nm (reference) wavelengths, respectively. Treatment with 7.3 μM of Sorafenib induced a statistically significant (*α_adj _= 0.017) inhibition of proliferation in SEM, RS4;11 and Jurkat cells, 0.73 μM Sorafenib in SEM cells as well. Results are displayed as mean +/- SD of three independent experiments.

Sorafenib inhibited cell cycle progression therby leading to a decreased cell proliferation. Cell cycle analysis exhibited an increase of SEM and Jurkat cells in G0/G1 phase which was accompanied by a reduction of cells in S- and M phase from 20.4%, 32.2% (DMSO) to 10.1%, 31.4% (0.73 μM) and 6.8%, 17.1% (7.3 μM) at 96 h, respectively. Cell cycle analyses of SEM cells are displayed in Figure 3A. Results for both cell lines are summarized in table 1. In addition, G0/G1 arrest was confirmed by western blot analysis in SEM and Jurkat cells (Figure 3B). Downregulation of CDK4 and CyclinD3 were detected 24 h after Sorafenib treatment at 7.3 μM. The protein levels of the CDK4 inhibitor p15^INK4 ^increases, but in contrast the protein expression of CDK2 inhibitor p27^KIP1 ^decrease in SEM cells, whereas Sorafenib did not affected the CDK inhibitors in Jurkat cells.

**Table 1:** Results of cell cycle analyses in SEM and Jurkat cells

		SEM			Jurkat		
		
Time	Phase	DMSO	0.73 μM	7.30 μM	DMSO	0.73 μM	7.30 μM
**24 h**	**G0/G1**	84.6%	87.2%	85.4%	70.4%	72.8%	61.9%
	**S**	8.3%	4.2%	8.4%	6.8%	11.7%	18.2%
	**M**	7.1%	8.6%	7.2%	22.8%	15.5%	19.9%

**48 h**	**G0/G1**	79.6%	93.5%	86.3%	72.2%	57.3%	83.1%
	**S**	9.9%	2.3%	3.8%	13.3%	14.7%	6.7%
	**M**	10.5%	4.2%	9.9%	14.5%	28.0	10.2%

**72 h**	**G0/G1**	84.0%	95.9%	91.6%	75.2%	69.3%	83.0%
	**S**	8.7%	2.4%	6.2%	14.0%	18.3%	10.5%
	**M**	7.3%	1.7%	2.2%	10.8%	12.4%	6.5%

**96 h**	**G0/G1**	79.6%	89.9%	93.2%	67.8%	68.6%	82.9%
	**S**	10.7%	4.2%	3.3%	13.5%	17.0%	11.6%
	**M**	9.7%	5.9%	3.5%	18.7%	1.4%	5.5%

Furthermore, we evaluated metabolic activity by measuring mitochondrial dehydrogenases activity in cells using WST-1 assay. Incubating the cells with 7.3 μM Sorafenib resulted in a significant decrease of mitochondrial dehydrogenases activity in SEM, RS4;11 and Jurkat cells. Treatment with 0.73 μM Sorafenib induced a significant inhibition of metabolic activity in SEM cells but not in RS4;11 and Jurkat. Results of WST-1 assay after treatment with Sorafenib in SEM, RS4;11 and Jurkat after 48 h are shown in Figure 3C.

### Sorafenib inhibits Erk, mTOR and Akt

**Figure 4:**
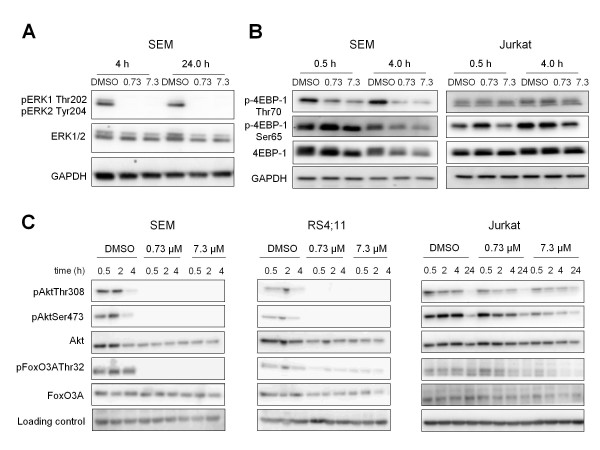
**Treatment with Sorafenib inhibits the Ras/Raf/Erk and PI3K/Akt/mTOR pathway**. Western blot experiments for serveral target proteins are displayed. ALL cell lines were treated with Sorafenib for the indicated time periods. Total cell lysates (25 μg) were separated by electrophoresis on an 8% SDS-Page gel. Membranes were incubated with the indicated antibodies against Erk1/2 and PI3K/AKT/mTOR signaling pathway-related proteins. GAPDH was used as control for equal loading. **(A) **Sorafenib induced down-regulation of pErk1/2 (Thr202 and Thr204) in SEM cells at 4 h and 24 h after treatment. **(B) I**nhibition of mTOR was detectable as a decrease of p-4EBP-1 (Thr70 and Ser65) protein in B- and T-ALL cells with 7.3 μM Sorafenib. **(C) **Sorafenib down-regulated the phosphorylation of Akt (Thr308 and Ser473) and reduced the phosphorylation of FoxO3A (Thr32) in SEM, RS4;11 and Jurkat cells.

Based on the observation that Sorafenib inhibits proliferation, we performed western blot analyses for Erk, 4-EBP-1, Akt and FoxO3A to characterize the effects of Sorafenib on Raf/Mek/Erk pathway and PI3K/Akt/mTOR pathway (Figure 4). Sorafenib induced a decrease in phosphorylated Erk1/2 (Thr202 and Tyr204) with 0.73 μM and 7.3 μM at 4 h and 24 h in SEM cells (Figure 4A). To investigate further the Sorafenib inhibition on the PI3K/Akt pathway, we examined downstream signaling of mTOR by analyzing the phosphorylation status of 4EBP-1. As shown in Figure 4B, treatment of 0.73 μM and 7.3 μM resulted in a suppression of p-4EBP-1 on both phosphorylation sites (Ser65 and Thr70) in SEM cells. In Jurkat cells mTOR signaling was exhibited by reduced phosphorylation of p-4EBP-1 on Ser65 and not modulated on Thr70 with 7.3 μM Sorafenib. Furthermore, incubation with Sorafenib for 0.5 h led to decreased levels of pAkt at both phosphorylation sites in SEM, RS4;11 and Jurkat cells. In line, pFoxO3AThr32 decreased. Whereas the inhibition of Akt were pronounced in SEM and RS4;11, they were less explicit in Jurkat cells (Figure 4C).

### Sorafenib acts synergistically in combination with cytostatics

**Figure 5:**
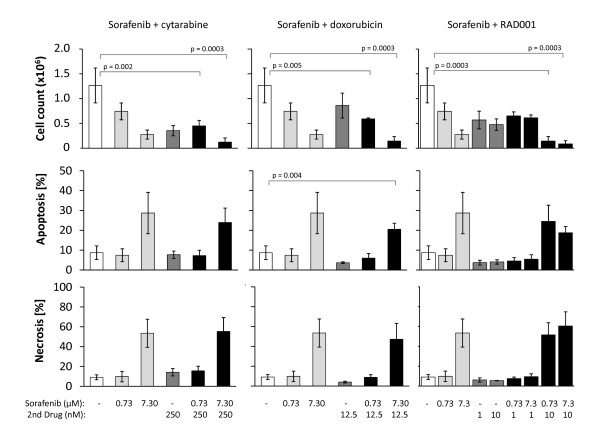
**Combination of Sorafenib with cytotoxic drugs inhibits cell proliferation and induces apoptosis and necrosis**. SEM cells were treated both separately and in combination with Sorafenib (0.73 μM or 7.3 μM) and with cytarabine (250 nM) or doxorubicin (12.5 nM) or RAD001 (1 nM or 10 nM) for 72 h. All drugs had effects on proliferation and rate of apoptosis and necrosis between treatment groups. Statistically significant effects were detected in regards to proliferation between all combination treatments vs. DMSO control and in regards to apoptosis between 7.3 μM Sorafenib and 12.5 nM doxorubicin vs. control (α_adj _= 0.006 for cytarbine and doxorubicine analyses; α_adj _= 0.003 for RAD001 analyses). Results are displayed as mean +/- SD of three independent experiments.

In order to detect and classify the effects of Sorafenib in combination with other cytostatics, a series of experiments were performed. Sorafenib (0.73 μM and 7.3 μM) was used in a total of eight simultaneous combinations with either 250 nM cytarabine, 12.5 nM doxorubicin, 1 nM or 10 nM RAD001 for up to 96 h. As treatment effects started to become apparent from time point 72 h (see Figure. 1), a more detailed analysis for SEM at 72 h is presented (Figure 5). Inhibition of cell proliferation of each of the combinations (Sorafenib + 2^nd ^Drug) compared to DMSO control reached statistical significance. Inhibition effects of single agent treatments vs. DMSO control, as well as combinations vs. single agents were detected, but did not reach statistical significance.

In addition, inhibitory treatment effects of all eight combinations on proliferation were classified to become "Bliss synergistic" ones with increasing concentrations of Sorafenib (the Bliss analyses are summarized in the additional file 1). This emphasizes a percentage increase in maximal inhibition and is above the expected strictly "Bliss additive" in nature effect, attributed just to the impact of Sorafenib-co-treatment with cytostatics. Further treatment effects on proliferation, apoptosis and necrosis are noticeable, but not verifiable, reflecting the feature of synergistic effects that mixture toxicities are detectable at low, statistically non-significantly acting concentrations of the single compounds.

## Discussion

Targeted therapy of leukemias with specific inhibitors has been shown to be effective and clinically well tolerated. In the present study, we have investigated the effect of the multikinase inhibitor Sorafenib in regards to influence on proliferation, apoptosis and necrosis in B- and T-lymphoblastic cells. Significant antiproliferative effects of Sorafenib were observed with 7.3 μM (a clinically achievable serum concentration) in all investigated cell lines. Inhibition of proliferation was also inducible with lower concentration (0.73 μM) in SEM cells. We could further demonstrate that Sorafenib induces caspase activation by cleavage of caspases 3 and 7 which results in cleavage of the nuclear protein PARP. Sorafenib has been previously shown to activate apoptosis and necrosis in various types of cancer [6,7,15-17]. In AML it was shown that treatment with Sorafenib activates the intrinsic apoptotic pathway by up-regulation of Bim associated with an increase of Bad, Bax and Bak proteins [8]. Further, it was demonstrated that Sorafenib-induced apoptosis resulted in down-regulation of Mcl-1, caspase activation and cytochrome c release in different cancer cells [6,18].

Whereas the effects of Sorafenib on Raf, Mek, Erk inhibition are well established in a variety of different cancers, its effects on the Akt signaling pathway is less clear. Since we and other have shown earlier, that the Akt pathway is activated in ALL cells, we aimed to detect Sorafenib effects on the PI3K/Akt/mTOR pathway. We demonstrated an inactivation of Akt after treatment with Sorafenib in SEM and RS4;11 achieving a complete disappearance of phosphorylated Akt. In Jurkat cells, that harbor a PTEN deletion, a marked decrease of pAkt levels (pAktSer473 and pAktThr308) occurred. In previous studies with several cancers, Sorafenib has not been shown to inhibit Akt phosphorylation [10,19,20]. Furthermore, it was demonstrated with biochemical assays, that Sorafenib is not a direct inhibitor of Akt [9]. In contrast, different studies indicated that Sorafenib induced an inhibition of STAT3 (phosphorylated signaltransducers and activators of transcription 3) that were associated with decreased levels of pAkt in glioblastoma cells, pancreatic cancer cell lines and neuroblastoma cells [16,21,22]. Moreover, we analyzed mTOR kinase activity by analyzing the phosphorylation sites of 4EBP-1. In SEM and Jurkat cells reduced levels of p-4EBP-1 (Ser65 and Thr70) were detected with 7.3 μM Sorafenib after 0.5 h treatment. In addition, we showed a decrease of phosphorylation of transcription factor FoxO3A as a result of an inhibition of Akt. Dephosphorylation of Akt leads to a relocalization of FoxO3A from cytoplasm into the nucleus and acts as transcription factor [23-25]. FoxO3A transcribes proapoptotic genes and cell cycle inhibitors such as *Bim-1, Bcl-6*, *Trail*, *p21Cip1*, *p27Kip1 *and *GADD45 *which proteins block cell cycle progression or induce apoptosis, respectively [26,27]. In our study, we showed that antiproliferative effects of Sorafenib are caused by cell cycle arrest as well as apoptosis. G0/G1 arrest was associated with a decrease of CyclinD3 and CDK4 as well as an increase of p15^INKB ^in SEM and Jurkat Sorafenib (0,73 μM) treated cells. In contrast, protein levels of CDK2 inhibitor p27^Kip1 ^were lower than in untreated SEM cells, indicating that CDK2 inhibition were affected not only by FoxO3A transcription factors. In comparison to SEM cells, protein expression of p27^Kip1 ^is lower and not affected in Jurkat cells.

Our results indicate that Sorafenib influences not only the Raf/Mek/Erk pathway but also the PI3K/Akt/mTOR signaling pathway. Ulivi P et al. showed that Sorafenib exhibit a strong anti-proliferative effect independently of Ras/Raf/Mek/Erk [21]. The mechanism by which Sorafenib inhibits Akt phosphorylation remains uncertain. Decreased levels of pAkt might be caused by inhibiting different upstream tyrosine kinases, as Sorafenib has also a potent activity against VEGFR, c-Kit, c-Raf and B-Raf [5]. Expression of VEGFR-2 has been demonstrated in haematopoetic stem cells. After ligand binding of VEGFR-2 and following autophosphorylation, the Ras/Raf/Mek/Erk pathway and the PI3K/Akt signaling cascade are activated [28]. In this context, we presume a cross-talk between both pathways that provide Akt inhibition after Sorafenib treatment in ALL cells. Recent studies described an interaction between MAPK and mTOR pathway [29,30]. These findings support our hypothesis that Sorafenib-induced inhibition of PI3K/Akt/mTOR as a result of blocking upstream several tyrosine kinases as well as Ras/Raf/Erk pathway.

Further, it is known that Sorafenib inhibits FMS-like tyrosine kinase 3 (FLT3). This receptor tyrosine kinase is mainly expressed in early myeloid and lymphoid progenitor cells and activates PI3K and Ras signal-transduction cascades [31]. Internal tandem duplication insertions in the juxtamembrane domain of FLT3 (FLT3-ITD) leads to constitutive activation of this receptor. FLT3-ITD are common in AML (25%) whereas this mutation occurs much less frequently in ALL (<1%) [32]. Additionally, it has been reported that mixed-lineage leukemia gene (MLL) rearrangement and hyperdiploid patients offer point mutations in the activation loop in the tyrosine kinase domain of FLT3 [33]. This mutation results in ligand-independent receptor dimerization, phosphorylation and constitutive activation of downstream signalling pathways and is more frequent in ALL (5-22%) [32,34]. SEM and RS4;11 are precursor B-ALL cell lines and carry the t(4;11) MLL-AF4 fusion protein. However, both cell lines are negative for FLT3-ITD.

In order to enhance antiproliferative effects, combination of cytostatic drugs is commonly a reasonable approach. Successful combination therapy might enhance the impact of inhibitors, allowing lower doses of cytostatic drugs and thereby reducing side effects. Different studies in patients with melanoma, hepatocellular carcinoma or non-small-cell lung cancer suggest that Sorafenib has the potential to be combined with a variety of cytotoxic drugs and targeted agents [35-37]. Here, we treated cells with 0.73 μM or 7.3 μM Sorafenib in combination with sub-IC50 concentrations of cytarabine, doxorubicin and the mTOR inhibitor RAD001. Our studies demonstrate that the *in vitro *effect of co-treatment with Sorafenib and cytostatics is "Bliss synergistic" in B-ALL cells regarding proliferation, apoptosis and necrosis as well. These results indicate that simultaneous application of Sorafenib with the tested conventional cytotoxic drugs might lead to synergistic impact, exceeding the expected pure "additive" effect of individual compounds with independent action. The results obtained indicate that the effects of the mixtures of Sorafenib and cytostatics can be studied experimentally, even at low concentrations of the single components.

We suppose that pre-treatment with cytarabine might potentate the *in vitro *effect of Sorafenib and maximize apoptosis and necrosis rates. Integration of cytarabine is restricted and occurs mainly during the S-phase of cells. In our study, we demonstrated that Sorafenib induced cell cycle arrest by decreasing the proportion of cells in the S and M phase hindering the efficacy of cytarabine. Auclair *et al. *reported that Sorafenib induces G0/G1 arrest in AML cells [7]. Levis *et al*. elucidated that pre-treatment with chemotherapy induce synergistic interactions, whereas treatment of cells with the FLT3 inhibitor CEP-701 followed by cytarabine administration results in antagonistic effect in FLT3/ITD expressing leukemia cell lines [38]. In contrast, Zhang *et al. *demonstrated that combination of Sorafenib with cytarabine synergistically inhibits *in vitro *growth in AML cells [8]. Further studies are warranted to show whether or not pre-treatment of cytostatic drugs potentate synergistic effects in Sorafenib treated ALL cells.

Additionally, we investigated antiproliferative effects of Sorafenib in combination with RAD001, a mTOR inhibitor to enhance toxicity in ALL cells. It has been shown, that inhibition of the Ras/Raf/Mek/Erk and PI3K/Akt/mTOR pathways is more effective and induces synergistically effects [36,39]. Combination of Sorafenib with RAD001 was associated with a significant inhibition of ALL cell growth. Previous studies demonstrated that RAD001 caused G1 cell cycle arrest and did not induce apoptosis in different cancer cell lines [40-42]. Furthermore, it was reported that combination of RAD001 with the new RAF inhibitor LBT613 led to a significant decreased proliferation in glioblastoma cells [43]. Treatment with RAD001 and Sunitinib synergistically inhibited the proliferation of leukemia cells [44]. A previous report by Tamburini et al., 2008 demonstrated that RAD001 induced an up-regulation of phosphorylated Akt levels in AML cells [45]. These data suggest that rather a pre-treatment than concomitant treatment with RAD001 may enhance Sorafenib antiproliferative effects on ALL cells. However, additional studies are needed to evaluate the efficacy of combination treatments with Sorafenib and anticancer drugs in ALL.

## Conclusion

This study shows that the multikinase inhibitor Sorafenib blocks cell proliferation and induces apoptosis by cleavage of caspases 3, 7 and PARP in ALL cells. In addition, we could demonstrate that Sorafenib significantly inhibits the PI3K/Akt/mTOR pathway, which might be an important action mode besides the well known effects on the Ras/Raf/Mek/Erk pathway. Given that Sorafenib displays significant antileukemic activity *in vitro*, it might be a potential drug for a target therapy approach in ALL.

## Competing interests

The research project was in part supported financially by BayerHealthcare, Leverkusen, Germany. There are no other financial or non-financial competing interests.

## Authors' contributions

CS designed experiments, performed and analyzed data and wrote the manuscript; MD participated in the study design, data interpretation and edited the manuscript, SR participated in the study design and data interpretation. MS carried out some experiments; FA carried out some experiments, SL participated in the design of experiments and edited the manuscript, DE served as a collaborator and assisted with experimental design; AG performed statistical analysis and edited the manuscript; GF served as a collaborator and edited the manuscript, SB performed statistical analysis and edited the manuscript; OW served as a collaborator and edited the manuscript; LMN served as a collaborator and assisted with experimental design MF: contributed to experimental design and organization CJ designed experiments, analyzed data and participated in the design of the paper and critically revised it.

All authors read and approved the final manuscript.

## Pre-publication history

The pre-publication history for this paper can be accessed here:

http://www.biomedcentral.com/1471-2407/10/560/prepub

## Supplementary Material

Additional file 1 **Bliss analyses of Sorafenib and 2^nd ^Drug effects on SEM cells**. Analyses of synergism interaction effect of two mixed substances (Sorafenib and a cytostatic) using Bliss additivism model for effect classification into "synergistic"/"antagonistic"/"additive" is displayed. The difference (delta) between a theoretical expected (white bar) and the experimental measured (grey bar) inhibition effect of the mixture on proliferation is > 0, reflecting a synergistic, above the expected Bliss-"additive" in nature effect. Sorafenib demonstrates synergystic effects on proliferation inhibition when combined with cytarabine, doxorubicin and RAD001. Click here for file

## References

[B1] Thomas DA Faderl S O'Brien S Bueso-Ramos C Cortes J Garcia-Manero G Giles FJ Verstovsek S Wierda WG Pierce SA Shan J Brandt M Hagemeister FB Keating MJ Cabanillas F Kantarjian H Chemoimmunotherapy with hyper-CVAD plus rituximab for the treatment of adult Burkitt and Burkitt-type lymphoma or acute lymphoblastic leukemia Cancer 2006 106 1569 80 10.1002/cncr.21776 16502413

[B2] Ottmann OG Wassmann B Pfeifer H Giagounidis A Stelljes M Duhrsen U Schmalzing M Wunderle L Binckebanck A Hoelzer D Imatinib compared with chemotherapy as front-line treatment of elderly patients with Philadelphia chromosome-positive acute lymphoblastic leukemia (Ph+ALL) Cancer 2007 109 2068 76 10.1002/cncr.22631 17429836

[B3] Vignetti M Fazi P Cimino G Martinelli G Di Raimondo F Ferrara F Meloni G Ambrosetti A Quarta G Pagano L Rege-Cambrin G Elia L Bertieri R Annino L Foa R Baccarani M Mandelli F Imatinib plus steroids induces complete remissions and prolonged survival in elderly Philadelphia chromosome-positive patients with acute lymphoblastic leukemia without additional chemotherapy: results of the Gruppo Italiano Malattie Ematologiche dell'Ad Blood 2007 109 3676 8 10.1182/blood-2006-10-052746 17213285

[B4] Wilhelm S Chien D BAY 43-9006: preclinical data Curr Pharm Des 2002 8 2255 7 10.2174/1381612023393026 12369853

[B5] Wilhelm S Carter C Lynch M Lowinger T Dumas J Smith RA Schwartz B Simantov R Kelley S Discovery and development of sorafenib: a multikinase inhibitor for treating cancer Nat Rev Drug Discov 2006 5 835 44 10.1038/nrd2130 17016424

[B6] Yu C Bruzek LM Meng XW Gores GJ Carter CA Kaufmann SH Adjei AA The role of Mcl-1 downregulation in the proapoptotic activity of the multikinase inhibitor BAY 43-9006 Oncogene 2005 24 6861 9 10.1038/sj.onc.1208841 16007148

[B7] Auclair D Miller D Yatsula V Pickett W Carter C Chang Y Zhang X Wilkie D Burd A Shi H Rocks S Gedrich R Abriola L Vasavada H Lynch M Dumas J Trail PA Wilhelm SM Antitumor activity of sorafenib in FLT3-driven leukemic cells Leukemia 2007 21 439 45 10.1038/sj.leu.2404508 17205056

[B8] Zhang W Konopleva M Shi Y McQueen T Harris D Ling X Estrov Z Quintas-Cardama A Small D Cortes J Andreeff M Mutant FLT3: a direct target of sorafenib in acute myelogenous leukemia J Natl Cancer Inst 2008 100 184 98 10.1093/jnci/djm328 18230792

[B9] Wilhelm SM Carter C Tang L Wilkie D McNabola A Rong H Chen C Zhang X Vincent P McHugh M Cao Y Shujath J Gawlak S Eveleigh D Rowley B Liu L Adnane L Lynch M Auclair D Taylor I Gedrich R Voznesensky A Riedl B Post LE Bollag G Trail PA BAY 43-9006 exhibits broad spectrum oral antitumor activity and targets the RAF/MEK/ERK pathway and receptor tyrosine kinases involved in tumor progression and angiogenesis Cancer Res 2004 64 7099 109 10.1158/0008-5472.CAN-04-1443 15466206

[B10] Strumberg D Preclinical and clinical development of the oral multikinase inhibitor sorafenib in cancer treatment Drugs Today (Barc) 2005 41 773 84 10.1358/dot.2005.41.12.937959 16474853

[B11] Metzelder S Wang Y Wollmer E Wanzel M Teichler S Chaturvedi A Eilers M Enghofer E Neubauer A Burchert A Compassionate use of sorafenib in FLT3-ITD-positive acute myeloid leukemia: sustained regression before and after allogeneic stem cell transplantation Blood 2009 113 6567 71 10.1182/blood-2009-03-208298 19389879

[B12] Bliss CI The toxicity of poisons applied jointly ANNALS OF APPLIED BIOLOGY 1939 26 585 615 10.1111/j.1744-7348.1939.tb06990.x

[B13] Buck E Eyzaguirre A Brown E Petti F McCormack S Haley JD Iwata KK Gibson NW Griffin G Rapamycin synergizes with the epidermal growth factor receptor inhibitor erlotinib in non-small-cell lung, pancreatic, colon, and breast tumors Mol Cancer Ther 2006 5 2676 84 10.1158/1535-7163.MCT-06-0166 17121914

[B14] Grimme HL ARBTFMBWaSM Combined Effects of Environmental Pollutants in Ecotoxicology: Biometrical Models as Concepts for Prediction and their Experimental Proof UWSF - Z. Umweltchem. Ökotox 12 226 234 10.1007/BF03038215

[B15] Zhang W Konopleva M Ruvolo VR McQueen T Evans RL Bornmann WG McCubrey J Cortes J Andreeff M Sorafenib induces apoptosis of AML cells via Bim-mediated activation of the intrinsic apoptotic pathway Leukemia 2008 22 808 18 10.1038/sj.leu.2405098 18200035

[B16] Yang F Brown C Buettner R Hedvat M Starr R Scuto A Schroeder A Jensen M Jove R Sorafenib induces growth arrest and apoptosis of human glioblastoma cells through the dephosphorylation of signal transducers and activators of transcription 3 Mol Cancer Ther 2010 9 953 62 10.1158/1535-7163.MCT-09-0947 20371721 PMC2852467

[B17] Huang S Sinicrope FA Sorafenib inhibits STAT3 activation to enhance TRAIL-mediated apoptosis in human pancreatic cancer cells Mol Cancer Ther 2010 9 742 50 10.1158/1535-7163.MCT-09-1004 20197401 PMC3281304

[B18] Rahmani M Davis EM Bauer C Dent P Grant S Apoptosis induced by the kinase inhibitor BAY 43-9006 in human leukemia cells involves down-regulation of Mcl-1 through inhibition of translation J Biol Chem 2005 280 35217 27 10.1074/jbc.M506551200 16109713

[B19] Liu L Cao Y Chen C Zhang X McNabola A Wilkie D Wilhelm S Lynch M Carter C Sorafenib blocks the RAF/MEK/ERK pathway, inhibits tumor angiogenesis, and induces tumor cell apoptosis in hepatocellular carcinoma model PLC/PRF/5 Cancer Res 2006 66 11851 8 10.1158/0008-5472.CAN-06-1377 17178882

[B20] Bonelli MA Fumarola C Alfieri RR La Monica S Cavazzoni A Galetti M Gatti R Belletti S Harris AL Fox SB Evans DB Dowsett M Martin LA Bottini A Generali D Petronini PG Synergistic activity of letrozole and sorafenib on breast cancer cells Breast Cancer Res Treat 2010 124 79 88 10.1007/s10549-009-0714-5 20054642

[B21] Ulivi P Arienti C Amadori D Fabbri F Carloni S Tesei A Vannini I Silvestrini R Zoli W Role of RAF/MEK/ERK pathway, p-STAT-3 and Mcl-1 in sorafenib activity in human pancreatic cancer cell lines J Cell Physiol 2009 220 214 21 10.1002/jcp.21753 19288493

[B22] Chai H Luo AZ Weerasinghe P Brown RE Sorafenib downregulates ERK/Akt and STAT3 survival pathways and induces apoptosis in a human neuroblastoma cell line Int J Clin Exp Pathol 2010 3 408 15 20490331 PMC2872747

[B23] Vogt PK Jiang H Aoki M Triple layer control: phosphorylation, acetylation and ubiquitination of FOXO proteins Cell Cycle 2005 4 908 13 15917664 10.4161/cc.4.7.1796

[B24] Van Der Heide LP Hoekman MFM Smidt MP The ins and outs of FoxO shuttling: mechanisms of FoxO translocation and transcriptional regulation Biochem J 2004 380 297 309 10.1042/BJ20040167 15005655 PMC1224192

[B25] Serra V Markman B Scaltriti M Eichhorn PJA Valero V Guzman M Botero ML Llonch E Atzori F Di Cosimo S Maira M Garcia-Echeverria C Parra JL Arribas J Baselga J NVP-BEZ235, a dual PI3K/mTOR inhibitor, prevents PI3K signaling and inhibits the growth of cancer cells with activating PI3K mutations Cancer Res 2008 68 8022 30 10.1158/0008-5472.CAN-08-1385 18829560

[B26] Greer EL Brunet A FOXO transcription factors at the interface between longevity and tumor suppression Oncogene 2005 24 7410 25 10.1038/sj.onc.1209086 16288288

[B27] Fu Z Tindall DJ FOXOs, cancer and regulation of apoptosis Oncogene 2008 27 2312 9 10.1038/onc.2008.24 18391973 PMC2819403

[B28] Cross MJ Dixelius J Matsumoto T Claesson-Welsh L VEGF-receptor signal transduction Trends Biochem Sci 2003 28 488 94 10.1016/S0968-0004(03)00193-2 13678960

[B29] Molhoek KR Brautigan DL Slingluff CLJ Synergistic inhibition of human melanoma proliferation by combination treatment with B-Raf inhibitor BAY43-9006 and mTOR inhibitor Rapamycin J Transl Med 2005 3 39 10.1186/1479-5876-3-39 16255777 PMC1289294

[B30] Sengupta S Schiff R Katzenellenbogen BS Post-transcriptional regulation of chemokine receptor CXCR4 by estrogen in HER2 overexpressing, estrogen receptor-positive breast cancer cells Breast Cancer Res Treat 2009 117 243 51 10.1007/s10549-008-0186-z 18807177 PMC2728144

[B31] Stirewalt DL Radich JP The role of FLT3 in haematopoietic malignancies Nat Rev Cancer 2003 3 650 65 10.1038/nrc1169 12951584

[B32] Armstrong SA Mabon ME Silverman LB Li A Gribben JG Fox EA Sallan SE Korsmeyer SJ FLT3 mutations in childhood acute lymphoblastic leukemia Blood 2004 103 3544 6 10.1182/blood-2003-07-2441 14670924

[B33] Taketani T Taki T Sugita K Furuichi Y Ishii E Hanada R Tsuchida M Sugita K Ida K Hayashi Y FLT3 mutations in the activation loop of tyrosine kinase domain are frequently found in infant ALL with MLL rearrangements and pediatric ALL with hyperdiploidy Blood 2004 103 1085 8 10.1182/blood-2003-02-0418 14504097

[B34] Small D FLT3 mutations: biology and treatment Hematology Am Soc Hematol Educ Program 2006 178 84 17124058 10.1182/asheducation-2006.1.178

[B35] Takimoto CH Awada A Safety and anti-tumor activity of sorafenib (Nexavar) in combination with other anti-cancer agents: a review of clinical trials Cancer Chemother Pharmacol 2008 61 535 48 10.1007/s00280-007-0639-9 18026728

[B36] Newell P Toffanin S Villanueva A Chiang DY Minguez B Cabellos L Savic R Hoshida Y Lim KH Melgar-Lesmes P Yea S Peix J Deniz K Fiel MI Thung S Alsinet C Tovar V Mazzaferro V Bruix J Roayaie S Schwartz M Friedman SL Llovet JM Ras pathway activation in hepatocellular carcinoma and anti-tumoral effect of combined sorafenib and rapamycin in vivo J Hepatol 2009 51 725 33 10.1016/j.jhep.2009.03.028 19665249 PMC2970800

[B37] Richly H Schultheis B Adamietz IA Kupsch P Grubert M Hilger RA Ludwig M Brendel E Christensen O Strumberg D Combination of sorafenib and doxorubicin in patients with advanced hepatocellular carcinoma: results from a phase I extension trial Eur J Cancer 2009 45 579 87 10.1016/j.ejca.2008.10.039 19101137

[B38] Levis M Pham R Smith BD Small D In vitro studies of a FLT3 inhibitor combined with chemotherapy: sequence of administration is important to achieve synergistic cytotoxic effects Blood 2004 104 1145 50 10.1182/blood-2004-01-0388 15126317

[B39] Wang Z Zhou J Fan J Qiu S Yu Y Huang X Tang Z Effect of rapamycin alone and in combination with sorafenib in an orthotopic model of human hepatocellular carcinoma Clin Cancer Res 2008 14 5124 30 10.1158/1078-0432.CCR-07-4774 18698030

[B40] Ikezoe T Nishioka C Bandobashi K Yang Y Kuwayama Y Adachi Y Takeuchi T Koeffler HP Taguchi H Longitudinal inhibition of PI3K/Akt/mTOR signaling by LY294002 and rapamycin induces growth arrest of adult T-cell leukemia cells Leuk Res 2007 31 673 82 10.1016/j.leukres.2006.08.001 17007924

[B41] Haritunians T Mori A O'Kelly J Luong QT Giles FJ Koeffler HP Antiproliferative activity of RAD001 (everolimus) as a single agent and combined with other agents in mantle cell lymphoma Leukemia 2007 21 333 9 10.1038/sj.leu.2404471 17136116

[B42] Park S Chapuis N Bardet V Tamburini J Gallay N Willems L Knight ZA Shokat KM Azar N Viguie F Ifrah N Dreyfus F Mayeux P Lacombe C Bouscary D PI-103, a dual inhibitor of Class IA phosphatidylinositide 3-kinase and mTOR, has antileukemic activity in AML Leukemia 2008 22 1698 706 10.1038/leu.2008.144 18548104

[B43] Hjelmeland AB Lattimore KP Fee BE Shi Q Wickman S Keir ST Hjelmeland MD Batt D Bigner DD Friedman HS Rich JN The combination of novel low molecular weight inhibitors of RAF (LBT613) and target of rapamycin (RAD001) decreases glioma proliferation and invasion Mol Cancer Ther 2007 6 2449 57 10.1158/1535-7163.MCT-07-0155 17766837

[B44] Ikezoe T Nishioka C Tasaka T Yang Y Komatsu N Togitani K Koeffler HP Taguchi H The antitumor effects of sunitinib (formerly SU11248) against a variety of human hematologic malignancies: enhancement of growth inhibition via inhibition of mammalian target of rapamycin signaling Mol Cancer Ther 2006 5 2522 30 10.1158/1535-7163.MCT-06-0071 17041096

[B45] Tamburini J Chapuis N Bardet V Park S Sujobert P Willems L Ifrah N Dreyfus F Mayeux P Lacombe C Bouscary D Mammalian target of rapamycin (mTOR) inhibition activates phosphatidylinositol 3-kinase/Akt by up-regulating insulin-like growth factor-1 receptor signaling in acute myeloid leukemia: rationale for therapeutic inhibition of both pathways Blood 2008 111 379 82 10.1182/blood-2007-03-080796 17878402

