# Supplementary figures and images for: The multikinase inhibitor Sorafenib displays significant antiproliferative effects and induces apoptosis via caspase 3, 7 and PARP in B- and T-lymphoblastic cells
Source: BMC Cancer. 2010 Oct 15;10:560. doi: 10.1186/1471-2407-10-560 (PMC2972283; doi:10.1186/1471-2407-10-560)

## Additional file 1

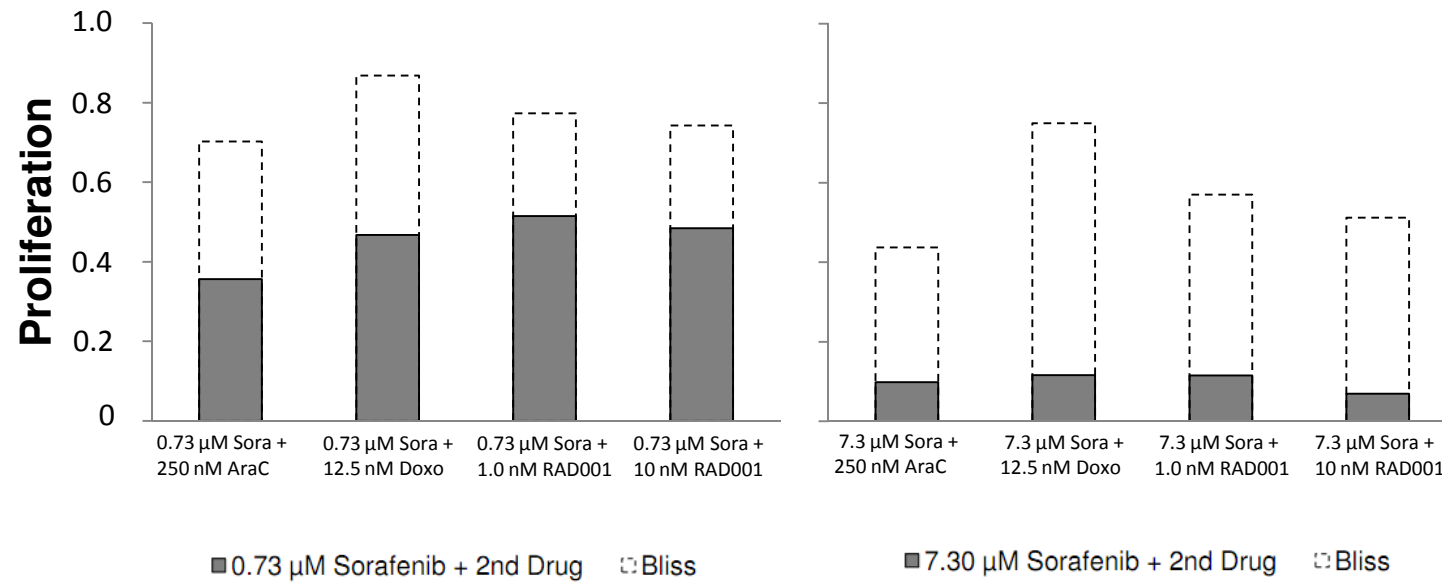

Supplement: Additional file 1 — Bliss analyses of Sorafenib and 2nd Drug effects on SEM cells. Analyses of synergism interaction effect of two mixed substances (Sorafenib and a cytostatic) using Bliss additivism model for effect classification into "synergistic"/"antagonistic"/"additive" is displayed. The difference (delta) between a theoretical expected (white bar) and the experimental measured (grey bar) inhibition effect of the mixture on proliferation is > 0, reflecting a synergistic, above the expected Bliss-"additive" in nature effect. Sorafenib demonstrates synergystic effects on proliferation inhibition when combined with cytarabine, doxorubicin and RAD001. [file 1471-2407-10-560-S1.PDF]
